# Two is Better Than One: Potentiating Cognitive Remediation With Aerobic Exercise to Improve Cognition in Schizophrenia With a Randomized Controlled Trial

**DOI:** 10.62641/aep.v53i4.1854

**Published:** 2025-08-05

**Authors:** Giulia Agostoni, Federica Repaci, Margherita Bechi, Irene Calzavara Pinton, Mariachiara Buonocore, Marco Spangaro, Jacopo Sapienza, Francesca Martini, Elisabetta D’Antoni, Beatrice Giglio, Federica Cocchi, Carmelo Guglielmino, Antonio Vita, Roberto Cavallaro, Giacomo Deste, Marta Bosia

**Affiliations:** ^1^Vita-Salute San Raffaele University, 20132 Milan, Italy; ^2^Department of Clinical Neurosciences, IRCCS San Raffaele Scientific Institute, 20127 Milan, Italy; ^3^Department of Molecular and Translational Medicine, University of Brescia, 25122 Brescia, Italy; ^4^University School for Advanced Studies (IUSS), 27100 Pavia, Italy; ^5^Department of Clinical and Experimental Sciences, University of Brescia, 25122 Brescia, Italy; ^6^Department of Mental Health and Addiction Services, ASST Valcamonica, 25040 Esine, Italy

**Keywords:** psychosis, rehabilitation, physical health, cognitive deficit, neurocognition

## Abstract

**Background:**

Cognitive impairment is a core feature of schizophrenia, for which pharmacological interventions have limited efficacy. Cognitive remediation (CR) is the gold standard for addressing cognitive deficits, yet its effect remains in the low-to-medium range, thus enhanced treatment approaches are needed. Emerging evidence supports the cognitive benefits of Aerobic Exercise (AE), suggesting that a combined intervention of AE and CR could lead to greater cognitive enhancements. This study aims at evaluating, with a randomized controlled trial, cognitive improvements following a combined intervention of CR+AE, compared to either CR or AE alone in patients diagnosed with schizophrenia.

**Methods:**

Sixty patients with schizophrenia were randomized into three groups (AE, CR, or CR+AE), and assessed for cognition, with the MATRICS Consensus Cognitive Battery at baseline, post-intervention, and at a 3-month follow-up.

**Results:**

CR+AE group showed significantly greater improvements in several domains including attention (*p* = 0.02), verbal learning (*p* = 0.03), and working memory (*p* = 0.04) compared to CR group, as well as processing speed (*p* = 0.002), verbal learning (*p* = 0.03), and working memory (*p* = 0.05) compared to AE group. At 3-months follow-up, evaluating CR+AE vs CR, further significant improvements were observed for social cognition (*p* = 0.01) in the CR+AE group, as well as for processing speed (*p* = 0.03) in the CR group.

**Conclusions:**

While preliminary, these findings suggest that a combined intervention of CR+AE allows greater improvements across core cognitive domains. In a wider perspective, this study also underscores the potential value of implementing aerobic exercise in rehabilitative approaches aimed at addressing cognitive dysfunction in schizophrenia.

## Introduction

Schizophrenia is a complex and severe mental disorder, often associated with poor functional outcome and impairments in cognitive, behavioral, emotional and social domains [1, 2, 3]. Cognitive impairment is considered a core feature of the illness, affecting around 80% of people with the disorder [4, 5]. Cognitive deficits, encompassing several domains, are major determinants of functional disruption and disability, also affecting personal autonomy, productivity, vocational status, social relationships and on the subjective quality of life [6]. These factors are also limiting the process of recovery in the context of psychiatric rehabilitation [7]. Given the significant economic burden associated with schizophrenia—estimated at over $261.6 billion per year in the U.S. [7] and approximately €8.5 billion per year in Italy [8] —it is crucial to identify effective intervention strategies.

Since current antipsychotic treatments provide minimal procognitive effects, research has increasingly focused on non-pharmacological interventions to mitigate cognitive impairments and improve functional outcome. Among them, Cognitive Remediation (CR) is a behavioral training-based intervention that has demonstrated efficacy in improving and preventing cognitive decline across multiple domains, with persistent effects on both cognition and functional outcome [9, 10, 11]. Also, the most recent European guidelines for schizophrenia treatment highlighted the efficacy of CR, suggesting that it is the best available intervention for improving cognition, daily functioning, and overall quality of life in this population [12]. However, studies evaluating CR efficacy indicate that the magnitude of improvement remains in the low-to-moderate range [13].

Among factors affecting the degree of response to CR, comorbid physical health conditions are worth of attention. Indeed, metabolic syndrome, affecting over 40% of people with schizophrenia [14, 15] is not only related to cognitive deficit in schizophrenia, but also appears to attenuate CR’s cognitive benefits [16]. This evidence suggests that standard CR protocols may not be sufficient and that prior or concomitant intervention targeting physical health should be recommended at least in selected patients [13].

In this view, aerobic exercise (AE) has emerged as a promising intervention, not only able to improve general physical health, but also with beneficial effects, through the stimulation of neuroplasticity brain effects, on cognitive performance, clinical symptoms, and socio-occupational functioning in the general population as well as in clinical ones, such as schizophrenia [17]. Recent studies have shown that AE promotes neuroplastic changes, including increased cortical thickness in specific brain regions [18], and enhances Brain-Derived Neurotrophic Factor (BDNF) levels, which support cognitive improvements, particularly in processing speed and cognitive flexibility [19]. Physical training in psychosis seems to improve positive and negative symptoms, cognitive disruption, social functioning and quality of life [20, 21, 22]. Moreover, AE increases cardiorespiratory fitness, metabolic health as well as neuroinflammatory markers [23], thus reducing the physical health problems associated with the illness [21].

Structural and functional imaging studies also revealed that AE induces a cascade of molecular processes and brain volume changes that lead to an improvement in cognitive functions as well as to a reduction of co-morbid conditions in patients with schizophrenia, such as Diabetes Mellitus Type II, hypertension, dyslipidemia, metabolic risk and inflammation [24, 25]. AE stimulates the release of neurotrophic factors (e.g., BDNF), which facilitates improvements in overall cognition [26, 27]. Also, AE seems to have positive effects on hippocampal neuroplasticity, increasing neurogenesis and synaptogenesis in response to cognitive stimulation [28] and stimulating the proliferation of neuronal precursor cells in the hippocampus [29].

As both CR and AE showed positive effects on improving cognition and functioning, are well tolerated and require relatively low costs for implementation in rehabilitation contexts, there is a significant clinical interest in better understanding the extent of their effectiveness and their potential as a combined treatment program. Preliminary results suggest the greater effectiveness of the combined intervention compared to the two interventions taken individually, although with mixed results [20, 22, 30]. A recent review summarizing the most relevant studies of the last ten years showed that the combination of CR and AE is effective in improving several cognitive domains, albeit with varying physical activity protocols [20]. Also, a pilot study by Nuechterlein and colleagues [21], assessing the impact of a 10-week program of cognitive training and physical exercise (CT&PE) in first-episode schizophrenia outpatients, showed significant greater improvements in CT&PE group for social cognition, working memory, processing speed and attention, compared to the group assigned to cognitive training alone (CTA). Furthermore, Dai and colleagues analyzing the efficacy of a short-term (8-week) aerobic exercise intervention alone (AEa) or in combination with computerized CR (AEc) in patients with schizophrenia, showed that AEc group significantly improved in processing speed and partial cognitive flexibility after the interventions. AEc patients also improved in processing speed and in the ability to inhibit cognitive interference at 12-week follow-up. Accordingly, Choi and colleagues [30] investigated the effects of either cognitive training (CT) focused just on processing speed and working memory and physical activity (PA) alone or a combined approach (CT+PA) in patients with schizophrenia, showing that working memory and processing speed improved post-intervention in all groups, with greater gains in the PA group. At a 3-month follow-up, only the CT+PA group showed significant improvements in working memory and processing speed. These results are encouraging, as they show that AE is a promising avenue for enhancing the effectiveness of CR programs. However, to date rehabilitative protocols are limited, and studies did not compared the effects of interventions combining CR and AE with both CR and AE.

While several studies explored the combination of CR and AE to improve cognition, definitive evidence of their superior efficacy compared to individual treatments is lacking. Our study aims to address this gap by providing a direct comparison of a combined CR+AE intervention with both CR and AE alone, offering robust data to support the need for integrated treatment strategies. Furthermore, the multicentric design of this study enhances its generalizability, while the inclusion of a 3-months follow-up evaluation provides evidence on the durability of cognitive improvements, further contributing to the innovation of the study. We hypothesize that participants in the CR+AE group will demonstrate significantly greater cognitive gains compared to those receiving CR or AE alone, thereby reinforcing the clinical relevance of integrated rehabilitation approaches for schizophrenia.

## Materials and Methods

### Participants

Sixty individuals diagnosed with schizophrenia (according to DSM 5 criteria) [31] were recruited from the Department of Clinical Neurosciences, Istituto di Ricovero e Cura a Carattere Scientifico (IRCCS) San Raffaele (Milan, Italy) and from the Department of Mental Health and Addiction Services, ASST Spedali Civili of Brescia (Brescia, Italy). Inclusion criteria were: age between 18 and 65, being able to give written informed consent. Exclusion criteria were: severe traumatic brain injury, neurological disorders, intellectual disability, alcohol or substance abuse in the preceding 6 months, and severe psychotic acutization in the preceding 3 months. All patients were on antipsychotic therapy and showed a good response to antipsychotic treatment as evaluated by the psychiatrist’s clinical judgment.

The protocol was approved by IRCCS San Raffaele Ethical Committee (ethics number 85/INT/2018) on 10/05/2018, following the principles of the Declaration of Helsinki, and subsequently authorized by the IRCCS San Raffaele Hospital 22/07/2019, thus ensuring the study’s ethical compliance, scientific validity, and methodological rigor, aligning with the highest standards of research integrity. The study was conducted from 22/07/2019 to 30/09/2023. All subjects provided informed consent, as they were deemed capable of providing informed consent based on the psychiatrist’s evaluation.

### Study Design

This is a multicenter, single-blinded, randomized controlled trial (RCT) study. All patients were randomized in a 1:1:1 ratio into 3 parallel treatment arms: we used a randomization table to randomly allocate participants either to the experimental intervention (CR+AE, N = 20) or to one of two active comparison groups (CR, N = 20; AE, N = 20). The total sample size (N = 60) was calculated with G*Power to detect medium effects (Cohen’s f = 0.25, alpha = 0.05) The sample of 20 subjects each will allow to meet 92% power.

### Interventions

The CR+AE and the CR groups participated to the CR intervention, consisting in three months of two 1-hour sessions per week using computer-assisted CR, performed with the Cogpack software [32]. This program targets different domain-specific neurocognitive exercises aimed at training the cognitive abilities that are altered in the patient. Sets of exercises were individually created for each patient, based on baseline neuropsychological performance. Most exercises are adaptive and the computer sets the level of difficulty, based on the patient’s performance during the course of the session. The program records the performance of the patient in each session, allowing patients to receive a feedback and therapists to track clinical progress. CR was administered by trained psychologists, whose role was to motivate patients and assist them in completing exercises and trying different strategies, without providing solutions to the exercises.

Participants randomized to the CR+AE and to the AE groups participated in 2 sessions of 1-hour aerobic exercise program for 3-months. Exercise sessions involved a group of 3 participants and included 15 minutes for stretching before and after the intervention. The exercise sessions included 45 minutes of exercise bike at moderate pace. Patients in the CR+AE group performed both Cognitive Remediation and Aerobic Exercise sessions on the same day, with a brief interval between activities.

All patients also received Treatment As Usual (TAU), which consists of a standard rehabilitation program, to balance treatment intensity as well as to ensure consistency in the level of care and control for treatment intensity. TAU consisted of routine, non-cognitive-focused care activities that align with standard psychiatric rehabilitation protocols. Specifically, TAU included psychoeducational sessions aimed at helping patients develop symptom management strategies, improve medication adherence, and enhance overall treatment compliance.

### Assessment

Trained psychologists evaluated all participants for cognition at baseline, after training, as well as at 3-month follow-up. Baseline data collected for each participant included also demographic information and clinical history.

Cognition was evaluated by the MATRICS Consensus Cognitive Battery (MCCB) [33], developed by the National Institute of Mental Health’s (NIMH’s), which provide an evaluation of key cognitive domains altered in schizophrenia. MCCB consists of 10 subtest: rail Making Test Part A (TMT); Brief Assessment of Cognition in Schizophrenia Digit Symbol-Coding (BACS SC); Hopkins Verbal Learning Test-Revised (HVLT-R); Wechsler Memory Scale Spatial Span (WMS-III SS); Letter-Number Span (LNS); Neuropsychological Assessment Battery Mazes (NAB); Brief Visuospatial Memory Test-Revised (BVMT-R); Category Fluency Test (Fluency); Mayer-Salovey-Caruso Emotional Intelligence Test Managing Emotions (MSCEIT ME); and Continuous Performance Test Identical Pairs (CPT-IP). The MCCB calculates T-scores for each subtest, which allows for the assessment of specific cognitive domains relevant to this study. These T-scores are then aggregated to generate composite scores for the key cognitive domains: Processing speed, Attention, Working memory, Verbal learning, Visual learning, Reasoning, and Social cognition.

### Data Analysis

Baseline differences between groups (CR+AE vs CR vs AE) were evaluated using analyses of variance (ANOVA) and Chi-Square test for categorical variables. We used the Least Significant Difference (LSD) test for post-hoc comparisons following ANOVA. The significance threshold was set at *p* = 0.05. Continuous data are presented as means ± standard deviations (SD) and categorical variables as frequencies and percentages.

To test the effects after the interventions (i.e., CR+AE vs AE and CR+AE vs CR), we run a series of analyses of covariance (ANCOVAs). We entered post-training measures as dependent variables, baseline measures as covariate, and treatment group (CR+AE vs AE and CR+AE vs CR) as grouping variable. Specifically, we included as dependent variables the after-training evaluation of MATRICS domains scores, Baseline measures, included in the models as covariates, were the before training evaluation of MATRICS domains scores. The significance threshold was set at *p* = 0.05.

To analyze the groups effects at 3-month follow-up, we used the same approach used for post-training, that is, we ran a series of ANCOVAs with follow-up measures as dependent variables, post-training measures as covariates, and treatment group (CR+AE vs AE and CR+AE vs CR) as grouping variable. The significance threshold was set at *p* = 0.05.

Analyses were conducted with STATISTICA Software for Windows, version 8 (StatSoft, Inc., Tulsa, OK, USA), IBM SPSS Statistics, version 28.0.1.0 (IBM Corp., Armonk, NY, USA), and Rstudio, version 2023.03 (RStudio, PBC, Boston, MA, USA).

## Results

### Baseline Characteristics of the Sample

At baseline, the sample showed a mean age of 36.19 years (±12.03 years), with a mean years of education of 11 years (±3.28 years), with 73.3% of the sample made up of male. As for clinical characteristics, the average age at onset was 21.33 years (±4.48 years), with a disease duration mean of 14.93 years (±11.27 years), and an average chlorpromazine equivalent dosage of 448.51 mg (±251.47 mg). Demographic, clinical, and cognitive measures stratified by groups (CR+AE, CR, AE) at baseline are reported in Table 1. The groups did not differ at baseline for any of the measures.

**Table 1.** **Sociodemographic and clinical characteristics of the groups at baseline**.

		CR (N = 20)	AE (N = 20)	CR+AE (N = 20)	ANOVA
		M (SD)	M (SD)	M (SD)	F, *p*
Age		35.83 (13.52)	39.60 (11.64)	32.13 (10.86)	F = 2.02, *p* = 0.13
Education		11.33 (2.63)	9.95 (4)	11.61 (2.88)	F = 1.55, *p* = 0.21
Age Onset		21.94 (4.54)	21.95 (4.72)	20.09 (4.10)	F = 1.24, *p* = 0.29
Illness duration		14.29 (12.26)	17.65 (11.67)	11.91 (10.03)	F = 1.39, *p* = 0.25
MATRICS					
	Processing speed	32.21 (15.67)	31.70 (14.26)	29.43 (2.58)	F = 0.23, *p* = 0.78
	Attention	40.08 (13.18)	32.70 (11.50)	33.88 (8.95)	F = 1.51, *p* = 0.23
	Working memory	40.42 (12.33)	35.85 (12.22)	34.48 (13.91)	F = 1.17, *p* = 0.31
	Verbal learning	39.53 (11.10)	41.80 (11.19)	35.30 (7.58)	F = 2.36, *p* = 0.10
	Visual learning	28.89 (10.40)	34.40 (7.19)	32.70 (13.69)	F = 1.29, *p* = 0.28
	Reasoning	36.58 (8.53)	37.85 (11.13)	37.30 (8.04)	F = 0.09, *p* = 0.91
	Social cognition	47.63 (14.55)	51.76 (10.82)	46.14 (14.73)	F = 0.84, *p* = 0.43

CR, Cognitive remediation; AE, Aerobic Exercise.

### Participant Compliance to Training

During the study, 18 participants (4 in the CR+AE group, 5 in the AE group, and 9 in the CR group) dropped out before post-training assessment, and 6 (1 in the CR+AE group, 1 in the AE group, and 4 in the CR group) dropped out before follow-up assessment. The drop-out rate after the intervention (30%) is in line with the average data for rehabilitation interventions in psychiatric populations, including schizophrenia [34]. Moreover, the dropout rate at the follow-up (40%) is lower that the medium rate observed in the literature, which usually shows a significant lost at follow-up that can overcome 70% [35]. In this study, the high dropout rate can be explained in the light of the period in which the study took place, as it started just few months before the beginning of the Covid-19 pandemic and continued throughout the pandemic phases.

### Post-training Effects

To test the cognitive improvements after trainings (i.e., CR+AE, CR, AE), we run a series of ANCOVAs.

**Fig. 1.**
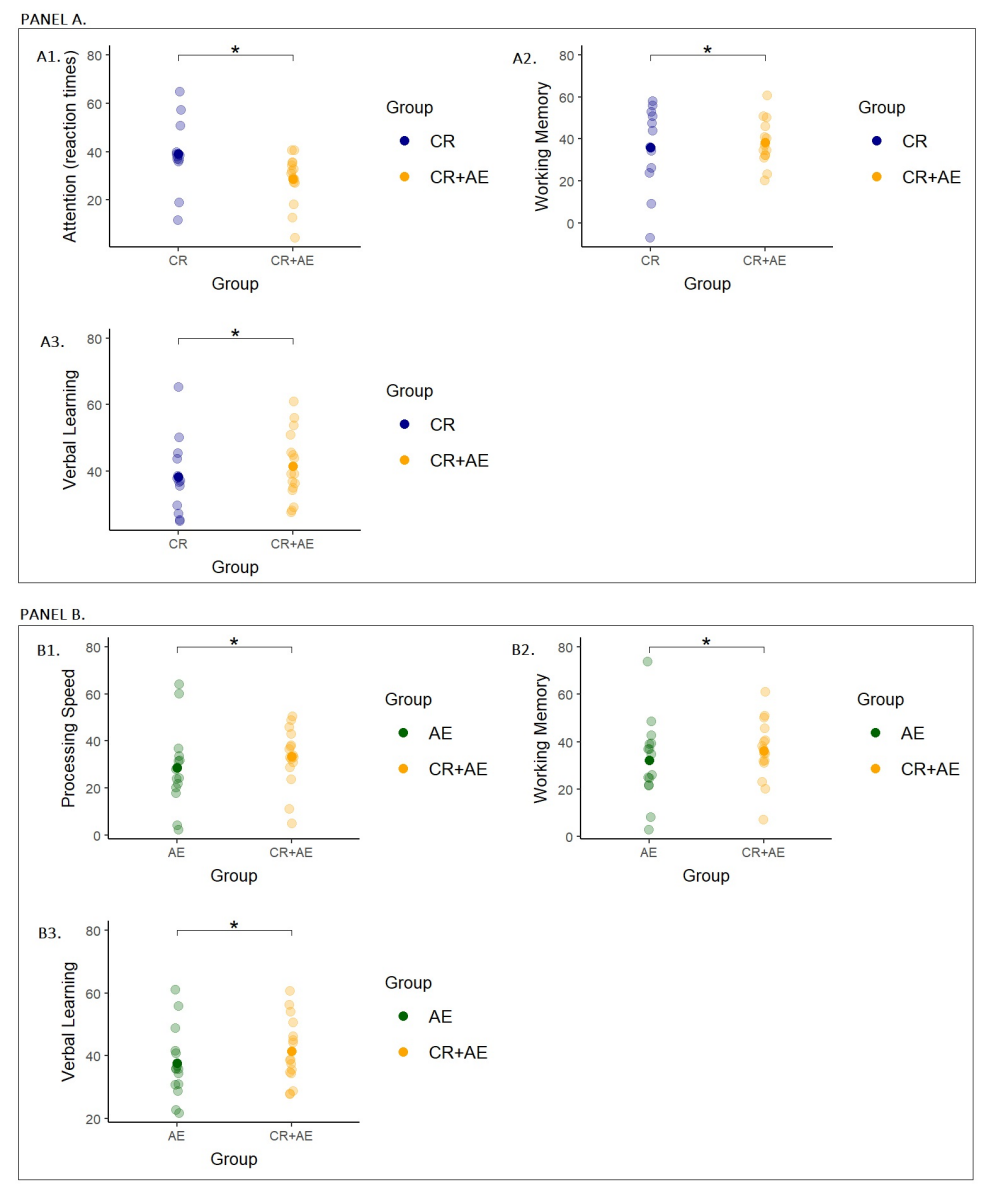
**Scatterplot of the estimated marginal means for the ANCOVAs on MATRICS measures at post-training for CR vs CR+AE and for AE vs CR+AE**. (Panel A) The figure shows the estimated marginal means for the ANCOVAs on the MATRICS Attention (A1), Working Memory (A2) and Verbal Learning (A3) scores at post-training. In each plot, the x-axis represents the group (CR vs CR+AE), while the y-axis represents the MATRICS measure. Estimated marginal means of the two groups are represented by the bold dots; bars represent the standard error of the mean and light-colored dots display the mean of the observed scores for each participant. * = *p* ≤ 0.05. (Panel B) The figure shows the estimated marginal means for the ANCOVAs on the MATRICS Processing Speed (B1), Working Memory (B2) and Verbal Learning (B3) scores at post-training. In each plot, the x-axis represents the group (AE vs CR+AE), while the y-axis represents the MATRICS measure. Estimated marginal means of the two groups are represented by the bold dots; bars represent the standard error of the mean and light-colored dots display the mean of the observed scores for each participant. * = *p* ≤ 0.05.

As for the comparison between CR+AE group and CR group, as shown in Panel A, results showed significantly greater improvements in the MATRICS domains of Attention (F = 6.85, *p* = 0.02), Working Memory (F = 4.41, *p* = 0.04) and Verbal Learning (F = 4.80, *p* = 0.03). Fig. 1A shows the distribution of scores obtained by the two groups in the significantly improved domains. The CR+AE group achieved significantly greater improvements in working memory, verbal learning skills, and attention, with a reduction in response time, compared to the CR group.

As for the comparison between CR+AE and AE groups, as shown in Panel B, results showed significantly greater improvements in the MATRICS domains of Processing Speed (F = 10.59, *p* = 0.002), Working Memory (F = 4.14, *p* = 0.05) and Verbal Learning (F = 4.69, *p* = 0.03). Fig. 1B shows the scores obtained by the two groups in these domains, evidencing that the CR+AE group achieved greater improvements in processing speed, working memory and verbal learning, compared to the AE group.

### Three-month Follow-up Effects

**Fig. 2.**
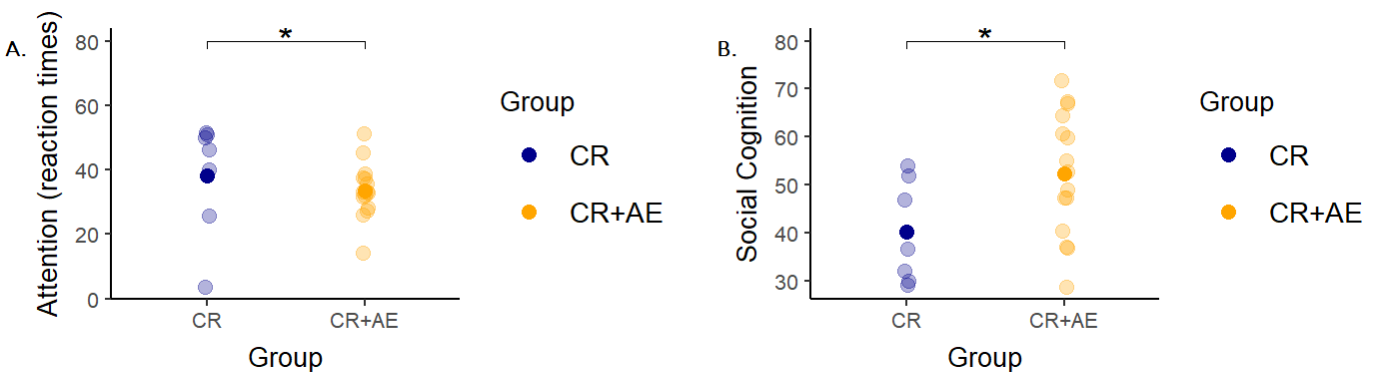
**Scatterplot of the estimated marginal means for the ANCOVAs on MATRICS measures at follow-up for CR vs CR+AE**. The figure shows the estimated marginal means for the ANCOVAs on the MATRICS Attention (A) and Social Cognition (B) scores at follow-up. In each plot, the x-axis represents the group (CR vs CR+AE), while the y-axis represents the MATRICS measure. Estimated marginal means of the two groups are represented by the bold dots; bars represent the standard error of the mean and light-colored dots display the mean of the observed scores for each participant. * = *p* < 0.05. Concerning the comparison between CR+AE vs AE groups at 3-month follow-up, results showed no significant improvement at T2 compared to T1, showing the stability of the improvements achieved after the intervention by the CR+AE group.

Results from the comparison between CR+AE and CR groups at T2 (i.e., 3-months follow-up) showed significant differences in the MATRICS domains of Processing Speed (F = 5.00, *p* = 0.03) and Social Cognition (F = 8.47, *p* = 0.01). The achieved cognitive improvements at T1 obtained by the CR+AE group remained stable in the other domains, as shown by the lack of significant differences from T1 to T2. Fig. 2 shows the distribution of the scores achieved by the two groups in the improved domains. The CR+AE group obtained, compared to the CR group, a significantly greater improvement at follow-up in social cognition, while the CR group achieved a greater performance in processing speed.

## Discussion

The severity of cognitive disruption in schizophrenia, as well as the evidence that pharmacological treatments have only a limited effect, has led to considerable research and clinical interest in non-pharmacological interventions aimed at improving cognitive deficits. In line with this view, this study innovatively aimed to fill an important gap in the literature by testing the efficacy of a combined intervention integrating CR and AE (i.e., CR+AE) compared to either CR or AE alone. Unlike previous studies, which often lacked direct comparisons between combined and individual interventions, this study provides strong evidence supporting the greater efficacy of CR+AE over each component alone. Additional strengths of this research include its multicenter design, which enhances the generalizability of findings, and the inclusion of a follow-up evaluation, which offers important insights into the durability of cognitive improvements achieved with the combined intervention.

Our results showed that a combined intervention integrating CR+AE is associated with greater gains in key cognitive domains compared to either CR or AE alone. Specifically, results from the comparison between CR+AE and CR showed greater improvements in several cognitive domains, namely attention, working memory and verbal learning, with a reduction in response time to the attention test. Regarding the comparison between CR+AE and AE, improvements after the intervention were observed in processing speed, working memory and verbal learning only in the CR+AE group. Our results are of particular significance, as the improved domains are those most impaired in patients with schizophrenia and are closely linked to difficulties in everyday tasks and activities. In the literature, there is still scattered evidence comparing the efficacy of CR+AE with both AE and CR alone. Our results are in line with previous evidence by Nuechterlein and Dai, which showed that a rehabilitative intervention, combining both CR and AE, has greater effects on core cognitive domains, such as processing speed, working memory, and attention, than either CR or AE alone [21, 30, 31]. In contrast, a previous study by Choi *et al*. [30], showed larger improvements in the group undergoing AE alone, compared to those participating in a combined rehabilitative program. However, differences may stem from the specific AE modalities used, which included treadmill, elliptical, rowing machines, stationary bikes, weight training, free weights, and outdoor activities, as well as the fact that the CR program focused only on processing speed and working memory domains. In contrast, our study adopted a more comprehensive approach, embracing a broader range of cognitive domains known to be disrupted in schizophrenia, and used a tailored approach, thus costumizing cognitive exercises based on the specific difficulties of each patient.

Overall, our results and the limited studies in the literature suggest that rehabilitative programs, combining CR and AE, may produce more significant cognitive gains than CR or AE, possibly due to the synergic effects of the interventions. To date, CR has been recognized in the most recent guidelines for schizophrenia treatment as the gold standard intervention for cognitive impairment. Our study adds another piece to the puzzle by showing that CR yields enhanced cognitive benefits when coupled with other non-pharmacological interventions, such as AE. Current evidence show that AE induces a cascade of molecular and brain volume changes, as well as modification in structural and functional brain plasticity, leading to an improvement in cognitive functions [28, 29]. Moreover, it has been suggested that both CR and AE rely on similar mechanisms, such as BDNF, thus their combination may produce a significant release of neurotrophic factors facilitating improvements in overall cognition. Another aspect that should be accounted concerns the hypothesis of an influence of metabolic status on patient’s response to CR. As a matter of fact, parameters related to metabolic syndrome, such as triglycerides, blood pressure and fasting glycaemia, proved to be associated with worst cognitive profile, suggesting that their improvement through AE could enhance the effects of CR [16]. Hence, incorporating physical exercise into a combined treatment protocol appears to be a promising strategy for improving cognitive abilities in schizophrenia.

Follow-up results also corroborated the effectiveness of CR+AE compared to the other groups. Data comparing CR+AE with CR showed significant differences at the 3-month follow-up (compared to after-treatment) in processing speed, and social cognition. CR+AE group showed an improvement in sociocognitive abilities, obtained after the end of the intervention, while CR group improved in processing speed abilities. Moreover, results showed that the improvement achieved by the CR+AE group after the intervention remained stable in all domains. Relative to the comparison of CR+AE vs AE, results showed no significant improvements at the follow-up, supporting the stability of the achieved cognitive gains. Our results align with previous scattered findings, demonstrating either continued improvements or sustained maintenance of the gains achieved by the CR+AE group even after treatment completion [30, 31]. These results are also in line with previous evidence on CR alone, which showed that the persistence of cognitive gains could be appreciated also at 5 and 10 years after the end of the treatment. In sum, by combining CR and AE, we anticipated a synergic effect, as aerobic exercise may have enhanced the neural pathways targeted by cognitive remediation, ultimately leading to greater cognitive gains. This combined approach has the potential to address both general cognitive deficits and specific cognitive domains targeted by CR protocols, resulting in greater cognitive improvements compared to either intervention alone.

Limitations should be acknowledged. Firstly, the small sample size and the drop rate observed in our study (30% after the intervention and 40% at the follow-up) which has have limited the inclusion of other confounding factors into the analyses. Furthermore, it is plausible that a higher intensity of aerobic activity, i.e., three sessions per week, might have augmented the efficacy of this intervention in combination with cognitive remediation. Nevertheless, the limited adherence to aerobic activity in this patient population, predominantly sedentary, would have posed a significant challenge, making it difficult to increase the frequency of aerobic exercise. Moreover, the absence of individualized exercise customization based on metrics such as maximum oxygen uptake and target heart rate, may have influenced the efficacy of aerobic exercise interventions across different fitness levels. Lastly, the lack of normality tests prior to performing ANOVA may impact the robustness of the results given the small sample size.

## Conclusions

Our study demonstrates that integrated interventions combining CR+AE offer superior benefits and greater cognitive improvements compared to CR or AE alone. Moreover, the cognitive gains persisted at 3-month follow-up, indicating durable effects, thus further supporting the value of this integrative approach. Although preliminary, these results are encouraging and highlight the potential value of implementing aerobic exercise in rehabilitative settings addressing cognitive dysfunction in schizophrenia. The promotion and adoption of such rehabilitative protocols in clinical settings is essential to provide effective and personalized treatments for those living with schizophrenia, ultimately contributing to improve functional outcome and quality of life. Future studies should incorporate additional elements, including educational and motivational interventions to enhance adherence to AE and promote a healthier lifestyle that integrates both physical activity and proper nutrition in patients with schizophrenia.

## Availability of Data and Materials

The datasets generated and analyzed during the current study are not publicly available due to restrictions imposed by participant consent and institutional ethical guidelines. Participants did not provide explicit consent for their data to be shared publicly. However, aggregated data or additional information may be made available upon reasonable request to the corresponding author, subject to ethical approval.
